# Structural and Functional Elucidation of Peptide Ts11 Shows Evidence of a Novel Subfamily of Scorpion Venom Toxins

**DOI:** 10.3390/toxins8100288

**Published:** 2016-09-30

**Authors:** Caroline M. Cremonez, Mohitosh Maiti, Steve Peigneur, Juliana Silva Cassoli, Alexandre A. A. Dutra, Etienne Waelkens, Eveline Lescrinier, Piet Herdewijn, Maria Elena de Lima, Adriano M. C. Pimenta, Eliane C. Arantes, Jan Tytgat

**Affiliations:** 1 Laboratório de Toxinas Animais, Departamento de Física e Química, Faculdade de Ciências Farmacêuticas de Ribeirão Preto, Universidade de São Paulo (USP), Ribeirão Preto 14040-903, São Paulo, Brasil; carolmarroni@yahoo.com.br (C.M.C.); ecabraga@fcfrp.usp.br (E.C.A.); 2 Laboratory for Medicinal Chemistry, Rega Institute for Medical Research, University of Leuven (KU Leuven), P.O. Box 922, Leuven 3000, Belgium; mohitosh_maiti@yahoo.com (M.M.); eveline.lescrinier@rega.kuleuven.be (E.L.); piet.herdewijn@rega.kuleuven.be (P.H.); 3 Toxicology & Pharmacology, University of Leuven (KU Leuven), Campus Gasthuisberg O&N2, P.O. Box 922, Leuven 3000, Belgium; steve.peigneur@pharm.kuleuven.be; 4 Laboratório de Venenos e Toxinas Animais, Departamento de Bioquímica e Imunologia, Instituto de Ciências Biológicas, Universidade Federal de Minas Gerais (UFMG), Belo Horizonte 31270-901, Brasil; jscassoli@gmail.com (J.S.C.); aledutra@gmail.com (A.A.A.D.); melenalima@icb.ufmg.br (M.E.d.L.); apimenta@icb.ufmg.br (A.M.C.P.); 5 Laboratory of Protein Phosphorylation and Proteomics, University of Leuven (KU Leuven), P.O. Box 922, Leuven 3000, Belgium; etienne.waelkens@med.kuleuven.be

**Keywords:** *Tityus serrulatus*, scorpion toxin, Ts11, neurotoxin, NMR, protein structure, ICK fold, electrophysiology, potassium channel

## Abstract

To date, several families of peptide toxins specifically interacting with ion channels in scorpion venom have been described. One of these families comprise peptide toxins (called KTxs), known to modulate potassium channels. Thus far, 202 KTxs have been reported, belonging to several subfamilies of KTxs (called α, β, γ, κ, δ, and λ-KTxs). Here we report on a previously described orphan toxin from *Tityus serrulatus* venom, named Ts11. We carried out an in-depth structure-function analysis combining 3D structure elucidation of Ts11 and electrophysiological characterization of the toxin. The Ts11 structure is highlighted by an Inhibitor Cystine Knot (ICK) type scaffold, completely devoid of the classical secondary structure elements (α-helix and/or β-strand). This has, to the best of our knowledge, never been described before for scorpion toxins and therefore represents a novel, 6th type of structural fold for these scorpion peptides. On the basis of their preferred interaction with voltage-gated K channels, as compared to all the other targets tested, it can be postulated that Ts11 is the first member of a new subfamily, designated as ε-KTx.

## 1. Introduction

Scorpion venom is a rich source of potassium channel blocking toxins (KTxs), which have been used in the structural and functional characterization of various voltage-gated potassium (Kv) channels [1]. 

Kv channels have received much attention because they are widespread in almost all tissue, and also due to the high diversity of Kv channels expressed in mammalian cells. They play key roles in the regulation of many physiological processes, including neurotransmitter release, immune response, heart rate, insulin secretion, neuronal excitability, epithelial electrolyte transport, smooth muscle contraction and cell proliferation [2]. 

Interestingly, scorpions have only developed one structural fold arsenal of toxins targeting Na^+^ and Cl^−^ channels, while repeatedly evolving several folds to capture diverse prey via Kv channels [2].

To date, scorpion venom peptides are known to adopt five different structural folds. Most of them contain a common core topology comprised of one or two short α-helices connected to a triple-stranded antiparallel β-sheet stabilized by three or four disulfide bonds [3]. For both classes of toxins—those acting on potassium channels (KTxs) and those acting on sodium channels (NaTxs)—the range of different folds is merely the variability of CSα/β and CSα/α topology [1]. 

Pimenta et al. [4] reported the primary sequences of three new short peptides from *Tityus serrulatus* venom (Tsv) previously known as TsPep1 (KPKCGLCRYRCCSGGCSSGKCVNGACDCS), TsPep2 (TVKCGGCNRKCCAGGCRSGKCINGKCQCY), and TsPep3, and henceforth named Ts11, Ts12 and Ts13 (following the nomenclature suggested by Cologna et al. [5]). These peptides are 29 amino-acid residues long, ranging from approximately 2900 to 3000 Da. They are highly reticulated by four disulfide-bridges, which make these peptides the most constrained structures of scorpion venom-derived peptides known to date, and a unique group of neurotoxins found in *Tityus serrulatus* venom containing the vicinal cysteines [6]. 

Based on alignment and size evidence, Ts11, Ts12 and Ts13 were previously classified as KTxs. However, no subfamily was specified since there was no functional study or structure to base the classification on. In spite of some sequence similarities within the *C*-terminal β-sheet of well-characterized toxins active in K^+^ channels, their biological function has not been clarified until now [4]. 

We hereby present the sixth structural fold to be adopted by scorpion venom peptides, highlighted by an knotting type fold, stabilized by four disulfide bridges and completely devoid of the classical secondary structure elements such as α-helix and β-strand. To the best of our knowledge, this fold has not been described thus far for scorpion toxins. Based on the functional characterization of both the voltage-gated potassium channel (Kv) and voltage-gated sodium channel (Nav), and in depth structure-function analyses, we propose that Ts11 can be seen as member of a new KTx subfamily, designated as ε-KTx1.1. A highly homologous peptide, Ts12, was also purified and characterized as far as we were able. The results hereof are presented as Supplementary Material.

## 2. Results 

### 2.1. Isolation of Native Ts11 and Biochemical Characterization

**Figure 1:**
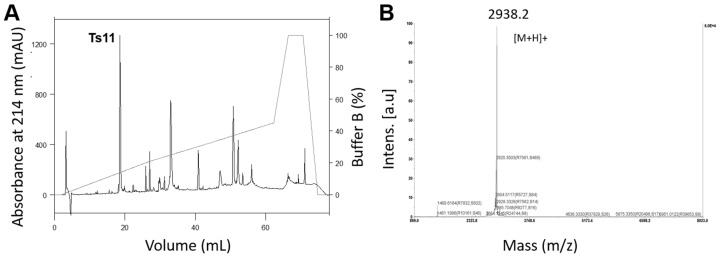
Ts11 isolation procedure and molecular mass determination. (**A**) Reversed-phase fast protein liquid chromatography (RP-FPLC) profile of fraction XIIA from *Tityus serrulatus* venom on a C8 column; (**B**) Mass spectra of Ts11 was obtained through MALDI-TOF mass.

Ts11 was isolated using a combination of cation exchange chromatography of *T. serrulatus* venom [7], followed by reversed-phase fast protein liquid chromatography (RP-FPLC) of fraction XIIA, as described in the experimental procedures section. The fraction XIIA was fractionated on a C8 column and afforded 17 chromatoghaphic peaks (Figure 1A). The second peak was identified as Ts11 by *N*-terminal sequencing and MALDI-TOF analysis (*m*/*z* 2938.2) (using alpha-Cyano-4-hydroxycinnamic acid (HCCA) matrix and reflectron positive ion mode (Figure 1B). Ts12 was isolated through reversed-phase high-performance liquid chromatography (RP-HPLC) of *T. serrulatus* venom on a C18 column (Supplemental Materials Figure S1A). The toxin was eluted in approximately 24 mL and its observed *m*/*z* was 2991.3 was confirmed by MALDI-TOF analysis (Supplemental Materials Figure S1B).

### 2.2. Structural Elucidation of Ts11

**Figure 2:**
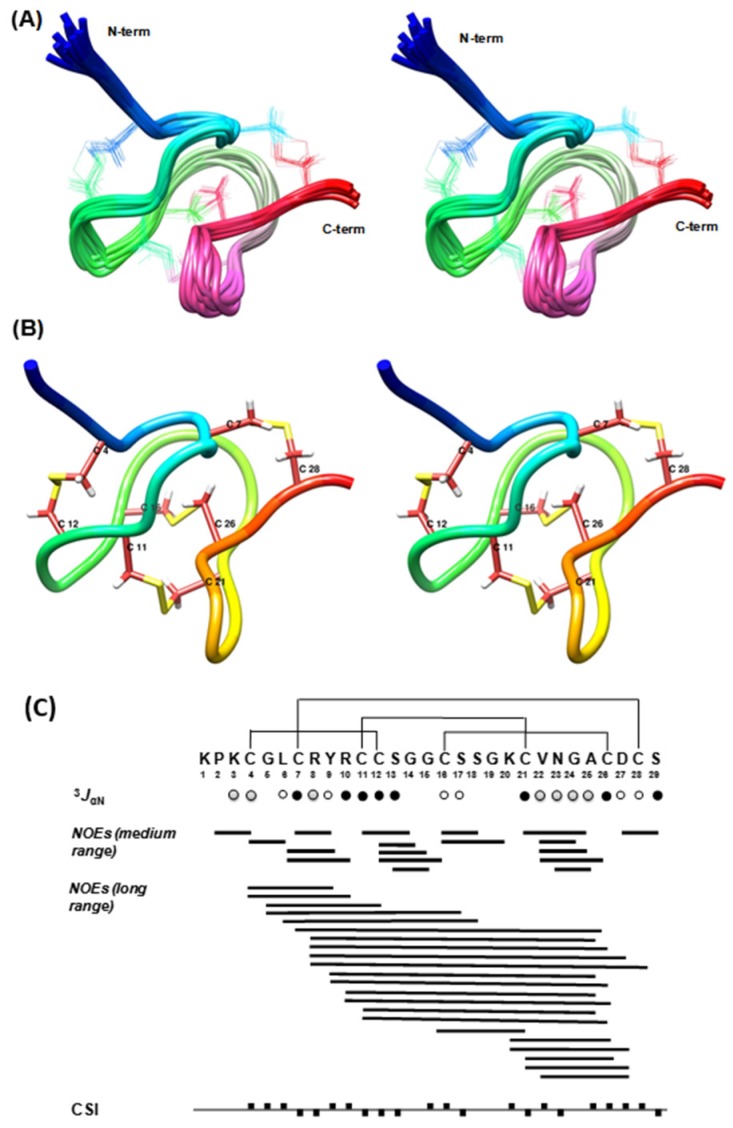
NMR solution structure of toxin peptide Ts11. (**A**) Stereoview of 15 final structures of Ts11 with superimposed backbone heavy atoms (N, CA, C’); (**B**) Stereo ribbon views of the closest-to-average structure of Ts11. Side chains of Cysteine residues are displayed along with their residue labels. Disulfide bonds are shown in yellow; (**C**) Amino acid sequence and disulfide connectivity of toxin peptide Ts11 along with a summary of medium and long range NOEs, ^3^*J*_HNHα_ (^3^*J*_αN_) couplings, and chemical shift index (CSI) for the Hα protons. 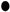 ^3^*J*_HNHα_ < 6 Hz, 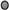 ^3^*J*_HNHα_ = 6–8 Hz, 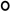 ^3^*J*_HNHα_ > 8 Hz. The filled squares above and below the horizontal line represent CSI values of +1 and −1 respectively.

Figure 2 displays the stereo images of the final ensemble of 15 superimposed structures (Figure 2A) and the minimum-energy closest-to-average structure (Figure 2B) of scorpion peptide Ts11. The peptide structures are well defined with backbone and heavy atom RMSD of 0.44 and 1.03 respectively over the entire chain, and Figure 1C shows the observed medium and long range NOEs that were used for structure determination, ^3^*J*_HNHα_, and chemical shift index (CSI) along with the amino acid sequences of toxin peptide Ts11. The CSI and ^3^*J*_HNHα_ coupling values suggest the likely absence of any *α*-helix and/or β sheet structure in the Ts11 peptide. 

The coordinates for 15 structures, NMR restraints and chemical shifts have been deposited in the RCSB Protein Data Bank with RCSB ID RCSB 103995, PDB ID 2MSF, and Biological Magnetic Resonance Bank BMRB with accession number: 25122.

NMR spectral analysis shows the formation of a single set of resonances for the Ts11, indicating that it adopts one type of structural form in solution. Resonance assignment was performed according to standard procedures as outlined by Wüthrich [8]. Complete sequence specific proton assignments were achieved by analyzing homo-nuclear two-dimensional (2D) spectra (DQF-COSY, TOCSY, and NOESY). Initially, the NH and Hα resonances of the individual spin systems (except Pro) were identified by analyzing the so-called “fingerprint” region of the DQF-COSY and TOCSY spectra and the remaining resonances of the spin systems were identified by following the so-called “TOCSY-tower”. Sequence specific assignments were achieved by linking each individual spin system via sequential inter-residue Hα*n*-HN (*n* + 1) cross-peaks in the “fingerprint” region of the NOESY spectrum. Partial carbon assignments were also performed by using ^1^H-^13^C HSQC spectra, which further reconfirmed most of the homo-nuclear proton assignments and clarified side chain proton assignments of other residues that were not resolved in the homo-nuclear 2D spectra. The geminal methylene protons were not assigned stereo-specifically and the NOE distance restraints involving these protons were used ambiguously during structure calculation in the Xplor-NIH program. 

The structural evaluation using PROCHECK demonstrates that none of the resulting structures have bad non-bonded contacts and most of the backbone dihedral angles are within the allowed regions of the Ramachandran plot (94.6% residues fall in the allowed region). Detailed structure determination statistics are provided in Table 1.

### 2.3. Functional Characterization of Ts11

**Figure 3:**
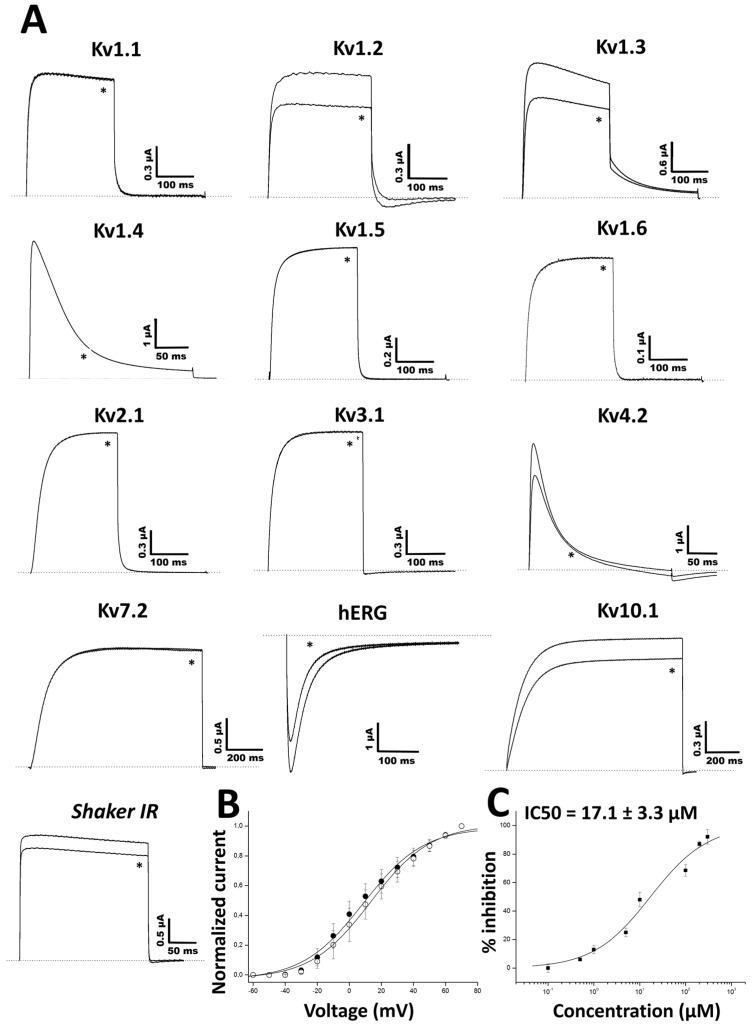
Electrophysiological study of Ts11 on voltage-gated potassium channels (Kv) expressed in Xenopus oocytes and measured using the two-electrode voltage-clamp. (**A**) Representative whole cell current traces on 13 different cloned voltage-gated potassium channels in the absence (control) and in the presence (*) of 3 μM native Ts11 (*n* ≥ 3); (**B**) Current-Voltage relationship on Kv1.3 in the absence (black circle, control) or in the presence (empty circle) of 5 μM Ts11 (*n* ≥ 3); (**C**) Dose-response curve of Ts11 on Kv1.3 (*n* ≥ 3).

The electrophysiological study of Ts11 was performed on voltage-gated sodium and potassium channels, Nav and Kv respectively. Both peptides were tested on different Kvs. Ts11, at 3 μM, showed a blocking effect on Kv1.2 (25%), Kv1.3 (27%), Kv4.2 (25%), Kv10.1 (15%), hERG (12%), and *Shaker* IR (10%) (Figure 3A).

To investigate the voltage-dependence of the channel activation, the activation curves were constructed using the normalized peak amplitudes of the I_tail_ values (Figure 3B). No significant alteration of properties of channel activation was observed since V_1/2_ values yielded 17.3 ± 0.6 and 17.6 ± 0.7 mV for the control and the toxin condition, respectively. The concentration-dependent inhibition of Kv1.3 currents by Ts11 revealed an IC_50_ value of 17.1 ± 3.3 μM (Figure 3C). Ts11 was also tested in higher concentrations on Kv1.2 (5 μM), hERG (10 μM) and Kv4.2 (5 μM) (data not shown), however no significant increase in blockage of these channels was observed. 

**Figure 4:**
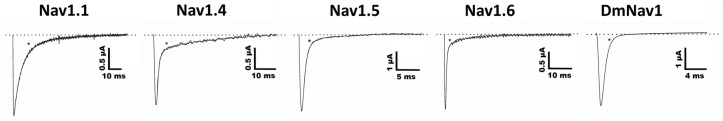
Electrophysiological study of Ts11 on voltage-gated sodium channels (Nav). Representative whole cell current traces in control and in the presence of native Ts11 (1 μM, number of cells ≥ 3) on 5 expressed voltage gated sodium channels, in the absence or presence (*).

Ts11 (1 μM) was also screened against a panel of 5 voltage-gated sodium channels (Nav1.1, Nav1.4, Nav1.5, Nav1.6 and the insect channel DmNav1) (Figure 4). The toxin was found to be inactive against these channels, at this concentration range, suggesting specificity towards Kv channels.

Ts12 at 3 μM showed blocking effect on Kv1.2 (5%), Kv1.3 (10%), Kv1.4 (20%), hERG (24%) and Shaker IR (27%). (Figure S2B).

## 3. Discussion

Ts11 has a unique feature in its structure: it is a short peptide of only 29 residues with two vicinal cysteines at the positions 11 and 12, adopting a highly constricted structure that is stabilized by 4 disulfide bonds. 

In an earlier study published by Pimenta et al. [4] it was reported that the enzymatic approach to described disulfide mapping for Ts11 (TsPep1) was unsuccessful due to the existence of two vicinal Cys residues and possibly due to the existence of a very compact TS11 structure. In this study, we have undertaken a solution state NMR study to investigate the four-disulfide linkages and to determine the three-dimensional structure of the Ts11 peptide. 

A closer look into the structure (Figure 2A,B) of Ts11 reveals that the peptide backbone adopts a very compact cysteine-knot like structure with three loops and one β-turn (V22-A25) at the *C*-terminal part. The structure is reinforced by four disulfide bonds (Cys^4^-Cys^12^, Cys^7^-Cys^28^, Cys^11^-Cys^21^, and Cys^16^-Cys^26^). All five glycine residues, as anticipated, are positioned in the β-turn and in the loops. The *N*-terminal end residues up to Cys^4^—notably containing two positively charged arginine residues—protrude out from the remaining compactly folded *C*-terminal head region like a screw tail. The exceptionality of the three-dimensional structure of Ts11 is its unique disulfide connectivity and the lack of any regular secondary structural units like α helices and/or β-strand, contrasting with what is commonly observed in the other scorpion toxins.

Ts11 was considered as a Kv blocker without altering the kinetics of channel gating. Although it is not a potent blocker of Kv channels, Ts11 does not target Nav channels, and therefore, it can be surmised that these toxins are representative blockers of Kv channels. However, it is possible that the Ts11 has other targets not yet identified.

Scorpion venom peptides are known to adopt five different structural folds. Most of them contain a common core topology comprised of one or two short α-helices connected to a triple-stranded antiparallel β-sheet stabilized by three or four disulfide bonds [3]. For scorpion venoms that affect Kv channel peptides, the fold known as the cystine-stabilized α/β (CSα/β) motif is the most abundant (around 208 peptides, according to Scorpktx [9]), and occurs in three distinct subfamilies: α-, β-and γ-KTxs [2,3,10,11]. It is important to note that Ts11 is not the first CSα/β motif-containing scorpion venom peptide that folds into a distinct three dimensional structure. For example, Ts16 and Maurotoxin are two CSα/β motif-containing peptides with a distinct cysteine pairing (C1-C5, C2-C4, C3-C6) and their structure only contains α-helices [12,13].

Another subfamily of KTxs with a different structural arrangement is the κ-KTx. These are purely helicoidal 3D structures named the cystine-stabilized helix-loop-helix (CSα/α) fold that consists of two short α-helices connected by a β-turn, stabilized by two disulfide bonds. To date, only 18 scorpion peptides adopting this fold have been discovered [11,14,15,16].

The δ-KTxs subfamily comprises all the Kunitz-type serine protease inhibitor scorpion toxins. These peptides exert both protease and potassium channel inhibiting properties. Conserved in this subfamily is the Kunitz-type structural fold with a double stranded antiparallel β-sheet flanked by an α-helix in both *C*-terminal and *N*-terminal segments. This fold is stabilized by three disulfide bridges: the first one connecting the *C*-terminal α-helix to one of the β-strands, and a second and third linking the *C*-terminal α-helix with the *C*-terminal and the *N*-terminal tail respectively [17,18]. 

The fourth subfamily of scorpion-venom peptides adopts the Inhibitor Cystine Knot (ICK) motif, with a triple stranded antiparallel β-sheet stabilized by 3 cystine linkages, and are predominantly found in cone snails and spider venoms [19,20]. The ICK is a structural motif that is shared with a large group of polypeptides having diverse primary structures and bioactivities. It is also found in peptides from evolutionarily distant organisms, such as fungi, plants, humans, marine mollusks and insects [19]. In scorpions, ICK peptides only represent minor venom components and target a limited number of receptors, such as Kv channels or ryanodine receptors [2]. 

The fifth fold is the disulfide-directed β-hairpin (DDH). It was previously suggested that the three disulfide bridge ICK fold is an elaboration of a simpler, ancestral two-disulfide fold coined the disulfide-directed β-hairpin (DDH) [2,20,21]. The ϕ-liotoxin, Lw1a is an example of a native peptide that adopts the previously hypothetical DDH fold. However, a recent critical revision by Undheim and colleagues noted that although both folds are related, it remains unclear whether or not the DDH fold really is the evolutionary precursor of the ICK motif. The fact that no other single-domain DDH peptides have been found in other organisms besides scorpions, suggests that the DDH is likely a derived ICK [20,21,22]

As in Ts11, vicinal cysteins also occur in γ-KTxs, δ-KTxs, λ-KTxs, λ-KTxs/calcines [2], and chlorotoxins. The ICK scaffold which we determined for Ts11 is in common with with λ-KTxs and λ-KTxs/calcine, although they possess only 3 disulfide bridges instead of 4.

**Figure 5:**
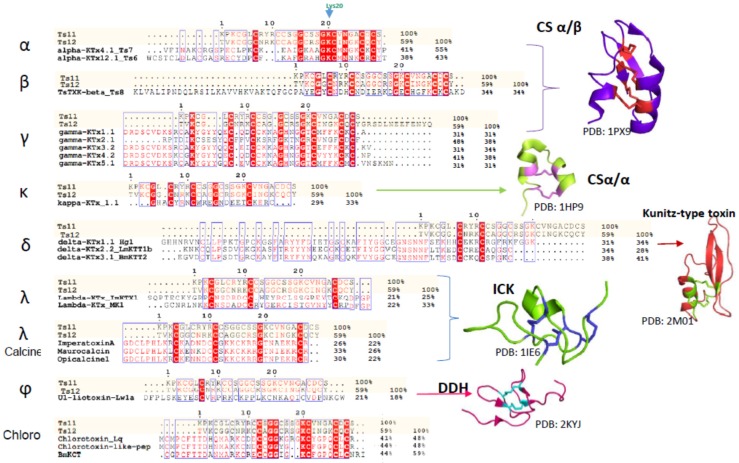
Amino acid sequence alignments and identities (%) among Ts11 and representatives of α-β-γ-κ-δ-λ-KTx-subfamilies, λ-KTx/calcine toxins, ϕ-liotoxin-Lw1a and chlorotoxins; and representatives of the five different structural folds adopted for scorpion toxins affecting KvsAlignment performed using ClustalW and MultAlin. The structures were created using Pymol and PDB database.

An extensive primary sequence comparison of Ts11 with other described toxins was performed. We aligned Ts11 with several toxins selective for voltage-gated potassium channels to all the currently known classes: α-β-γ-κ-δ-KTx, λ-KTx (ImKTx1 and λ-MK1, which are two scorpion toxins sharing the ICK motif and are functionally characterized as Kv blockers [2]), λ-KTxs/calcine toxins (Imperatoxin A, Maurocalcin and Opicalcine1 which are scorpion toxins sharing the ICK motif and are active on ryanodine receptors), ϕ-liotoxins-Lw1a [20,21]) and additionally the chlorotoxins (which also have vicinal cysteines in common with Ts11) (Figure 5). Although Ts11 was previously classified as a KTx, this toxin shows less than 50% of identity with all the subfamilies of KTx, α-β-γ-κ-δ- KTx. Even for γ and δ-KTx, which possess vicinal cysteines like Ts11, the identity remains between 30% and 48%. We also compared Ts11 with ICK type scorpion toxins, λ-KTx and λ-KTx/calcine, which share identities between 20% and 30%. Ts11 poorly shares identity (21%) with ϕ-liotoxins-Lw1a. Chlorotoxins showed identity around 41%–44% with Ts11.

**Figure 6:**
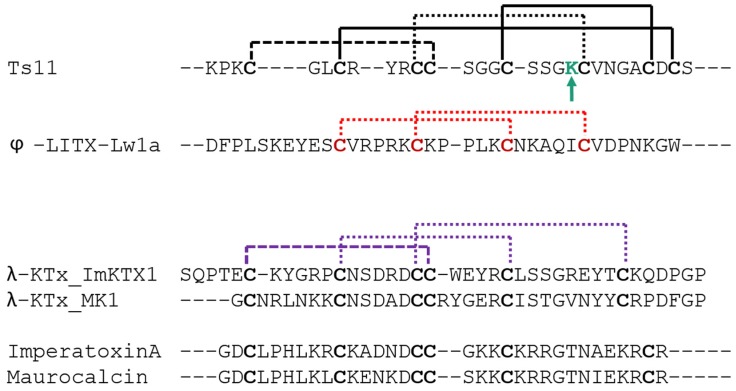
Comparison of the Ts11 with DDH-fold and ICK-fold toxins disulfide bond patterns. Disulfide patterns were compared with ϕ-Liotoxin-Lw1a (DDH motif), λ-KTxs and λ-KTx/calcine (three disulfide bridges ICK-type toxins, Imperatoxin A and Maurocalcin). Black lines represent the disulfide connectivity unique for Ts11. Red lines represent the DDH motif on ϕ-Liotoxin-Lw1a. Purple lines represent the disulfide connectivity on ICK-type toxins (λ-KTxs and λ-KTx/calcine). Long dashes: disulfide bond shared between Ts11 and ICK-type toxins. Dotted lines: disulfide connectivity shared between DDH motif and the ICK-type toxins. The green arrow indicates the positive charged residue of a possible dyad.

Further analyses highlight also the unique pattern of disulfide bridges of Ts11 when compared with other ICK type scorpion toxins (λ-KTx and λ-KTx/calcine) and with DDH-fold toxin (ϕ-liotoxins-Lw1a). The new pattern of disulfide connectivity in Ts11 can be regarded as novel organization of ICK type scorpion toxins, conserving elements from the typical ICK-fold scorpion toxins and from the DDH-fold, the evolutionary precursor of ICK motif that are stabilized by 3 and 2 disulfide bonds respectively [21] (Figure 6).

## 4. Conclusions

Many peptide toxins obtained from animal venoms have proved to be valuable tools for the elucidation of the pharmacological, physiological and structural features of their pharmacological receptors. 

Unravelling molecular determinants by which animal toxins are able to recognize a receptor or channel, and a detailed examination of their folds, provides several interesting research avenues in terms of protein engineering and therapeutic potential. Together, these tools can offer potential for altering pharmacological selectivity, specificity and potency of these toxins, making them a unique source of lead compounds and templates from which agents of specific therapeutic value may be designed and generated [1]. 

Ts11 tertiary structure obtained through solution NMR showed an ICK-type scaffold lacking the classical secondary structures, such as α-helix or β-strands, which, to the best of our knowledge has never been described thus far. Ts11 presents itself as a Kv blocker with unique structural features. Based on the novel scaffold of Ts11 and its high similarity with Ts12, we propose that these peptides are the first members of a sixth structural fold adopted by scorpion venom peptides. On the basis of a functional analysis evidencing these toxins as preferential Kv blockers as compared to all the other targets tested, and due to the poor percentage of identity with the other KTxs, we suggest that they can be regarded as the first members of a new subfamily of KTxs, named as ε-KTx. Therefore, Ts11 and Ts12 are named ε-KTX 1.1, and 1.2, respectively. 

## 5. Experimental Section

### 5.1. Ts11 and Ts12 Isolation Procedures

Ts11 was isolated by the fractionation of whole venom of *Tityus serrulatus* using a CM-cellulose-52 column adapted to a *FPLC Äkta Purifier UPC-10* (GE Healthcare, Uppsala, Sweden), as previously described by Cerni et al. [7]. This process afforded 18 fractions, named: I, II, III, IV, V, VIA, VIB, VII, VIIIA, VIIIB, IXA, IXB, X, XIA, XIB, XIIA, XIIB, XIII [7]. Fraction XIIA, containing the Ts11 peptide, was submitted to reversed-phase fast protein liquid chromatography (RP-FPLC) on an analytical C-8 column (Phenomenex-Aeriswidepore, 4.6 mm × 25 cm, 3.6 μm), previously equilibrated with solution A (0.1% trifluoroacetic acid, *v*/*v*), and eluted in a segmented gradient up to 100% of solution B (0.1% trifluoroacetic acid + 80% acetronitrile), at a flow rate of 1 mL/min and connected to a FPLC Äkta System equipment (GE Healthcare, Uppsala, Sweden). The isolated toxin was lyophilized and stored at −20 °C.

Ts12 was isolated by injecting whole venom of *T. serrulatus* in reversed-phase chromatography using the HPLC AKTA Explorer 100 system (GE Healthcare, Uppsala, Sweden), with the analytical column PepMap™ C18 (4.6 mm × 150 mm; Applied Biosystems, Foster City, CA, USA) which was previously equilibrated with 0.1% aqueous trifluoroacetic acid (TFA) (solution A). For each run, 50 mg of the lyophilized venom was dissolved in 1 mL of solution A. Samples were centrifuged, filtered and the supernatant applied to the column. Elution of the components was obtained by the following gradient system: 0–15 min, 0% B (solution B: 0.1% trifluoroacetic acid in acetonitrile); 15–50 min, 0%–30% B; 50–60 min; 30%–60% B. Flow was 1 mL/min and absorbance was monitored at 214 nm and 280 nm. Fractions were collected using an automated fraction collector Frac920 (GE Healthcare) on 96 deep well plates. Samples of interest were lyophilized and stored at −20 °C until required.

### 5.2. Biochemical Characterization of Toxins

The molecular mass of Ts11 was determined by MALDI-TOF mass spectrometry (4800 Analyzer, Applied Biosystems), alpha-Cyano-4-hydroxycinnamic acid (HCCA) matrix and analysis in reflectron positive ion mode. Amino acid sequencing was obtained by Edman’s degradation method [23], using PPSQ-33A equipment (Shimadzu Co., Kyoto, Japan), and sequences were aligned using the Mutalin interface [24], ClustalW [25] and Expasy (SIB Swiss Institute of Bioinformatics, Lausanne, Switzerland) [26]. 

Ts12 molecular mass was determined using AutoFlex III MALDI–TOF/TOF mass spectrometer (BrukerDaltonics, Billerica, MA, USA), alpha-Cyano-4-hydroxycinnamic acid (HCCA) matrix and analysis in reflectron positive ion mode. Peptide identity was confirmed by *N*-terminal sequencing using an automated PPSQ-21A protein sequencer (Shimadzu, Tokyo, Japan). 

### 5.3. Expression of Voltage-Gated Ion Channels in Xenopus Laevis Oocytes

For the expression of the voltage gated potassium channels (rK_V_1.1, rK_V_1.2, hK_V_1.3, rK_V_1.4, rK_V_1.5, rK_V_1.6, *Shaker* IR, rK_V_2.1, hK_V_3.1, rK_V_4.2, hERG, K_V_10.1) and the voltage gated sodium channels (rNa_V_1.1, rNa_V_1.4, hNa_V_1.5, mNa_V_1.6 and DmNav1) in *Xenopus* oocytes, the linearized plasmids were transcribed using the T7 or SP6 mMESSAGE-mMACHINE transcription kit (Ambion, Waltham, MA, USA). The harvesting of stage V–VI oocytes from anaesthetized female *X. laevis* frog has been previously described [27]. Oocytes were injected with 30–50 nL of cRNA at a concentration of 1 ng/nL using a micro-injector (Drummond Scientfic, Broomall, PA, USA). The oocytes were incubated in a solution containing (in mM): NaCl, 96; KCl, 2; CaCl_2_, 1.8; MgCl_2_, 2 and HEPES, 5 (pH 7.4), supplemented with 50 mg/L gentamycin sulfate.

### 5.4. Electrophysiological Recordings

Two-electrode voltage-clamp recordings were performed at room temperature (18–22 °C) using a Geneclamp 500 amplifier (Molecular Devices, Silicon Valley, CA, USA) controlled by a pClamp data acquisition system (Axon Instruments, Union City, CA, USA). Whole cell currents from oocytes were recorded 1–10 days after injection. Bath solution composition was ND96 (in mM): NaCl, 96; KCl, 2; CaCl_2_, 1.8; MgCl_2_, 2 and HEPES, 5 (pH 7.4) (in mM): NaCl, 2; KCl, 96; CaCl_2_, 1.8; MgCl_2_, 2 and HEPES, 5 (pH 7.4). Voltage and current electrodes were filled with KCl 3 M. Resistances of both electrodes were kept between 0.8 and 1.5 mΩ. The elicited currents were sampled at 1 kHz and filtered at 0.5 kHz (for potassium currents) or sampled at 20 kHz and filtered at 2 kHz (for sodium currents) using a four-pole low-pass Bessel filter. Leak subtraction was performed using a -P/4 protocol.

The K_V_1.1–K_V_1.6, K_V_2.1, K_V_3.1 and K_V_4.2 and *Shaker* IR currents were evoked by 250 ms depolarizations to 0 mV followed by a 250 ms pulse to −50 mV, from a holding potential of −90 mV. Current traces of hERG channels were elicited by applying a pulse from −90 mV to +40 mV for 2.5 s followed by a step to −120 mV for 2.5 s. The Kv 10.1 currents were evoked by 1 s depolarization to 0 mV, from a holding potential of −90 mV. Sodium current traces were evoked, from a holding potential of −90 mV, by 100 ms depolarization to 0 mV. In order to investigate the current-voltage relationship, current traces were evoked by 10 mV depolarization steps from a holding potential of −90 mV. 

To assess the concentration-response relationship of Ts11 on Kv1.3, data were fitted with the Hill equation: *y* = 100/[1 + (IC50/[toxin])*^h^*], where *y* is the amplitude of the toxin-induced effect, IC_50_ is the toxin concentration at half maximal efficacy, [toxin] is the toxin concentration and *h* is the Hill coefficient (Hill coefficient: 0.8). Current-voltage relationship was determined by 100 ms step depolarization between −90 and +70 mV, using 10 mV increments. All data represent at least 3 independent experiments (*n* = 3) and are presented as mean ± standard error.

### 5.5. NMR Spectroscopy

NMR spectra were recorded with a 1.7 mM solution (200 μL, pH 3.5) of the isolated native peptide Ts11 in H_2_O:D_2_O (9:1) mixture at 5 °C on a 600 MHz Bruker Avance II spectrometer equipped with a 5 mm TCI HCN Z-gradient cryoprobe. Spectra were processed using Topspin 2.1 (Bruker Biospin, Evere, West-Vlaanderen, Belgium) and analyzed by using CARA software (version 1.8.4, Vaughan, Ontario, ON, Canada) [28].

In the one-dimensional and two-dimensional spectra, the water signal was suppressed by using excitation sculpting with gradients [29]. The two-dimensional NOESY (mixing time 200 and 300 ms) was recorded with a sweep width of 7210 Hz in both dimensions, 64 scans, 2048 data points in *t*_2_, and 1024 free induction decays (FIDs) in *t*_1_.

A two-dimensional total correlation spectroscopy (2D-TOCSY) [30] was recorded with DIPSI2 sequence for mixing (mixing time 80 ms). A double quantum-filtered correlation spectrum (DQF-COSY) [31] was acquired using excitation sculpting with gradients for water suppression with a sweep width of 7210 Hz in both dimensions, 64 scans, 2048 data points in *t*_2_, and 1024 FIDs in *t*_1_. In the processing of two-dimensional spectra the data were apodized with a shifted sine-bell square function in both dimensions. Proton and carbon chemical shifts were calibrated by using external DSS signal as reference (0.000 ppm).

Natural abundance ^1^H, ^13^C heteronuclear single quantum correlation (^1^H-^13^C HSQC) spectrum was recorded on the natural abundance sample with sensitivity enhancement and gradient coherence selection optimized for selection of aliphatic CH groups (*J*_CH_ = 135 Hz) using 64 scans, 1024/2048 complex data points, and 12,072/7210 Hz spectral widths in *t*_1_ and *t*_2_ respectively. For the selection of aromatic CH groups 170 Hz was used for *J*_CH_ along with 32 scans, and 64/2048 complex data points.

### 5.6. Structural Constraints

Distance restraints were derived from cross-peak volumes of the NOESY spectrum recorded with 200 ms mixing time. Estimated interproton distances were derived using the isolated spin pair approximation, *r_ij_* = *r_ref_* (*v_ref_*/*v_ij_*)^1/6^ where *r*_ij_ is the estimated interproton distance, *r_ref_* is the fixed internal reference distance, and *v_ref_* and *v_ij_* are the NOE cross-peak volumes of the reference and estimated cross-peaks respectively. Average cross-peak volume of the geminal methylene proton pairs was used as reference volume which corresponds to the fixed reference distance of 1.8 Ǻ. Generally an experimental error of ±20% on the calculated interproton distances was used for upper and lower bounds. The ^3^*J*_HNHα_ coupling constants were measured from the one-dimensional proton spectrum recorded in H_2_O and then converted to dihedral restraints as follows: ^3^*J*_HNHα_ > 8 Hz, ϕ = −120° ± 30°; ^3^*J*_HNHα_ < 6 Hz; ϕ = −60° ± 30°; ω = 180° ± 30° to define the trans X-Pro conformation as confirmed by the observation of strong NOE interactions between Hα(*n*) and HD2, HD3(*n* + 1) Pro. 

### 5.7. Structure Calculations

All structure calculations were performed by using Xplor-NIH program, version 2.25 (National Institutes of Health Bethesda, Bethesda, MD, USA) [32]. A set of 100 structures was generated by torsion angle molecular dynamics, starting from an extended strand and by using only NMR-derived restraints, excluding any disulfide restraints. After the torsion angle molecular dynamics round [33], the majority of the structures had converged to very similar structures with similar total energies and with no violations of the NOE and dihedral restraints. In the initially derived structures all the disulfide bonds could be identified unambiguously by the observation of side chain proximity of eight Cys residues. Torsion angle molecular dynamics round was repeated for the second time including both the disulfide bond and NMR-derived restraints. The fifteen lowest energy structures from the second round were used for further refinement during a “gentle molecular dynamics” round in explicit water [34]. A box of water was constructed and optimized around selected structures obtained from the second torsion angle dynamics step. The final stage of refinement commenced with a 20 ps constant temperature molecular dynamics simulation at 300 K (20,000 steps of 0.001 ps) and was followed by a 200-step conjugate gradient energy minimization of the average structure of the last 10 ps of the 20 ps simulation. Structures were analyzed by using PROCHECK [35]. Visual representations were created by using UCSF Chimera software (version 1.9, University of California, San Francisco, CA, USA).

## Figures and Tables

**Table 1:** NMR structure determination statistics of scorpion toxin Ts11 for an ensemble of 15 structures. Values where applicable are represented by means ± S.D.

PROPERTIES	VALUES
**Total NOE Distance Restraints**	224
Intra residue	69
Sequential (׀*i* − *j*׀ = 1)	84
Medium range (2 ≤ ׀*i* − *j*׀ ≤ 4)	29
Long range (׀*i* − *j*׀ ≥ 5)	42
**Dihedral angle restraints**	13 (ϕ = 12, ω_X-Pro_ = 1)
**RMSD from the average structure (Ǻ)**	
Backbone atoms (N, C^α^, C’)	0.44 ± 0.08
All heavy atoms	1.03 ± 0.08
**RMS deviation from the idealized covalent geometry**	
Bond (Ǻ)	0.000007 ± 0.000000
Angle (°)	2.918 ± 0.110
Improper (°)	2.907 ± 0.311
**Ramachandran analysis (%)**	
Residues in favored regions	43.8
Residues in additional allowed regions	42.5
Residues in generously allowed regions	8.3
Residues in disallowed regions	5.4 (C4 & N23)

## Supplementary Materials

The following are available online at www.mdpi.com/2072-6651/8/10/288/s1. Figure S1: Ts12 isolation procedure and molecular mass determination. (**A**) Ts11 isolation. RP-HPLC profile of *Tityus serrulatus* venom (50 mg) on a C18 column (4.6 mm × 150 mm) equilibrated with 0.1% (*v*/*v*) of trifluoroacetic acid (TFA). Adsorbed proteins were eluted using acetonitrile concentration gradient from 0% to 60% of solution B (0.1% TFA in acetonitrile). Flow: 1 mL/min. Absorbance was monitored at 214 nm, at 25 °C; (**B**) Mass spectra of Ts12 was obtained thru MALDI-TOF mass spectrometry using HCCA matrix and reflectron positive ion mode, Figure S2: Electrophysiological study of Ts12 on Kv channels. Representative whole cell current traces in the absence (control) and in the presence (*) of 3 μM native Ts12 on 8 different cloned voltage-gated potassium channels. For each tested channel *n* ≥ 3.
