# Supplementary material for: Structural and Functional Elucidation of Peptide Ts11 Shows Evidence of a Novel Subfamily of Scorpion Venom Toxins
Source: Toxins (Basel). 2016 Sep 30;8(10):288. doi: 10.3390/toxins8100288 (PMC5086648; doi:10.3390/toxins8100288)
Supplement: Supplementary file 1 [file toxins-08-00288-s001.pdf]

Supplementary Materials: Structural and Functional Elucidation of Peptide Ts11 Shows Evidence of a Novel Subfamily of Scorpion Venom Toxins

Caroline M. Cremonez, Mohitosh Maiti, Steve Peigneur, Juliana Silva Cassoli, Alexandre A. A. Dutra, Etienne Waelkens, Eveline Lescrinier, Piet Herdewijn, Maria Elena de Lima, Adriano M. C. Pimenta, Eliane C. Arantes and Jan Tytgat

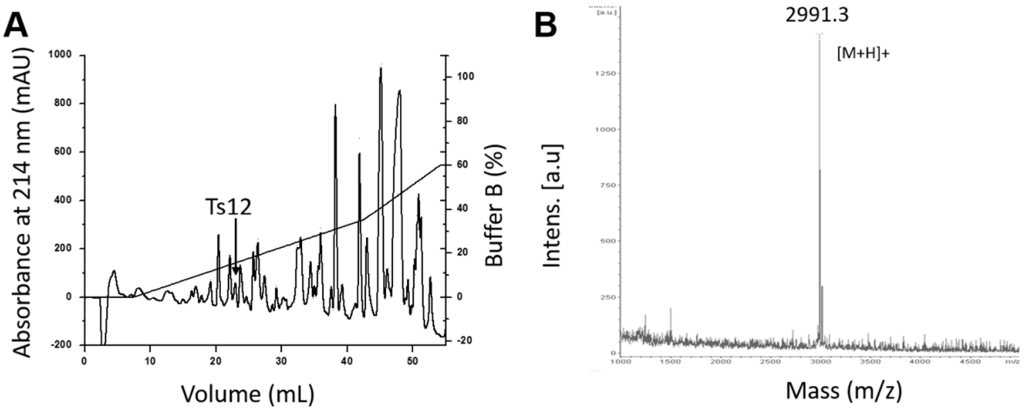

Figure S1. Ts12 isolation procedure and molecular mass determination. (**A**) Ts11 isolation. RP-HPLC profile of *Tityus serrulatus* venom (50 mg) on a C18 column (4.6 mm × 150 mm) equilibrated with 0.1% (*v*/*v*) of trifluoroacetic acid (TFA). Adsorbed proteins were eluted using acetonitrile concentration gradient from 0% to 60% of solution B (0.1% TFA in acetonitrile). Flow: 1 mL/min. Absorbance was monitored at 214 nm, at 25 °C; (**B**) Mass spectra of Ts12 was obtained through MALDI-TOF mass spectrometry using HCCA matrix and reflectron positive ion mode.

Ts12 at 3 μM showed blocking effect on Kv1.2 (5%), Kv1.3 (10%), Kv1.4 (20%), hERG (24%) and *Shaker* IR (27%). (Supplmentary Materials Figure S2).

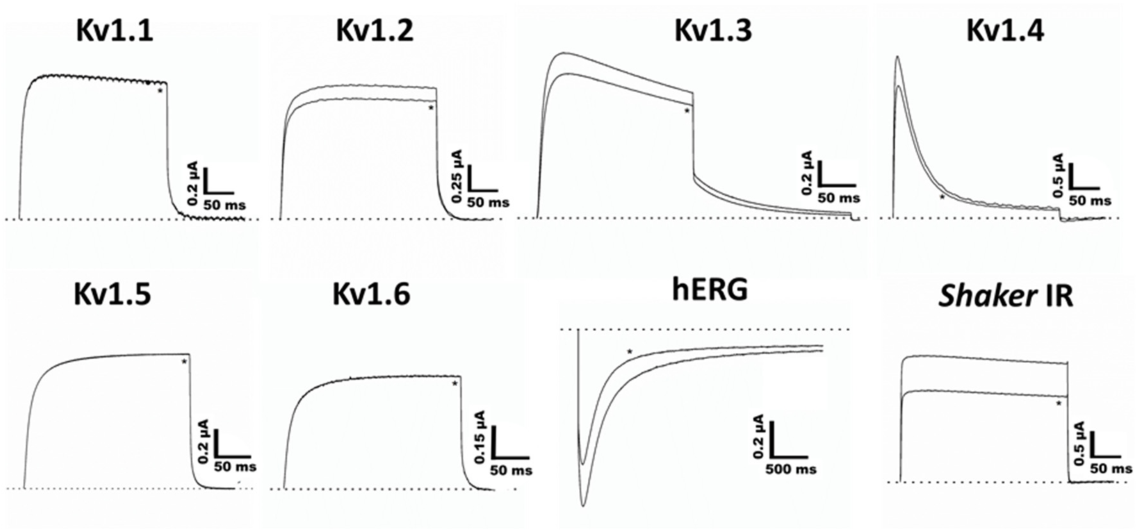

Figure S2. Electrophysiological study of Ts12 on Kv channels. Representative whole cell current traces in the absence (control) and in the presence (*) of 3 μM native Ts12 on 8 different cloned voltage-gated potassium channels. For each tested channel $n \geq 3$.
